# Fabrication of 3D Collagen-Based Decellularized Biological Scaffolds Using Human Wharton's Jelly-Derived Mesenchymal Stem Cells With Differentiation Potential Toward Chondrocytes

**DOI:** 10.1155/sci/9953810

**Published:** 2025-09-11

**Authors:** Fatemeh Masjedi, Zahra Heidari, Kamran Hosseini, Shahrokh Zare, Anahid Safari, Davood Mehrabani, Elmira Jalilian, Negar Azarpira, Zahra Khodabandeh

**Affiliations:** ^1^Nephro-Urology Research Center, Shiraz University of Medical Sciences, Shiraz, Iran; ^2^Shahid Chamran Hospital, Shiraz University of Medical Sciences, Shiraz, Iran; ^3^Shiraz Neuroscience Research Center (SNRC), Shiraz University of Medical Sciences, Shiraz, Iran; ^4^Stem Cells Technology Research Center, Shiraz University of Medical Sciences, Shiraz, Iran; ^5^Clinical Neurology Research Center, Shiraz University of Medical Sciences, Shiraz, Iran; ^6^Department of Oncology, Faculty of Medicine, University of Alberta, Edmonton, Canada; ^7^Department of Ophthalmology and Visual Sciences, Illinois Eye and Ear Infirmary, University of Illinois at Chicago, Chicago, USA; ^8^Transplant Research Center, Shiraz University of Medical Sciences, Shiraz, Iran; ^9^Netherlands Institute for Neuroscience, Royal Netherlands Academy of Arts and Sciences, Amsterdam, Netherlands

**Keywords:** biological scaffolds, chondrocyte, decellularization, human Wharton's jelly-derived mesenchymal stem cells, regenerative medicine

## Abstract

**Background:** Stem cell-based regenerative approaches have been developed to treat osteoarthritis (OA) and repair cartilage defects. In the present study, we fabricated a three-dimensional (3D) collagen-based decellularized biological scaffold using human Wharton's jelly-derived mesenchymal stem cells (hWJ-MSCs) and analyzed its recellularization and subsequent differentiation potential toward chondrocytes.

**Methods:** MSCs were isolated from human Wharton's jelly, characterized by flow cytometry, and differentiated toward osteogenic and adipogenic lineages. hWJ-MSCs were cultured in a 3D collagen scaffold. After the matrix was deposited by the cells, the scaffold was decellularized, and new hWJ-MSCs were cultured and differentiated into chondrocytes. The efficiency of the decellularization process was assessed using hematoxylin and eosin (H&E) staining, DNA quantification, scanning electron microscopy (SEM), and Raman spectroscopy. Immunohistochemical and transcriptional evaluation of chondrogenic markers, including collagen type II, aggrecan, and osteonectin, was performed.

**Results:** Prepared decellularized scaffolds showed very low levels of nucleic materials compared to intact ones. The integrity and efficiency of the decellularization process were confirmed using SEM. Moreover, a comparison of Raman spectra of intact and decellularized scaffolds demonstrated a remarkable reduction in carbohydrate, lipid, and DNA content. Three weeks after recellularization in the presence of chondrogenic medium, the immunoreactivity and expression levels of specific chondrocyte markers, including collagen type II, aggrecan, and osteonectin, significantly increased compared to negative controls.

**Conclusion:** hWJ-MSCs and their use in fabricating nucleic acid-free 3D collagen-based scaffolds represent a promising in vitro model for investigating how the extracellular matrix (ECM) contributes to specific cell microenvironments. Decellularized ECM can also be utilized to develop novel, cell-free biomedical products for regenerative medicine.

## 1. Introduction

Osteoarthritis (OA), the most common form of arthritis, results in progressive reductions in the volume of extracellular matrix (ECM) in joint cartilage, ultimately affecting joint function [1]. Treatment strategies for OA are typically inefficient and focused on reducing symptoms. Despite various surgical techniques, such as stimulating self-repair of the joint [2] and endoprosthetic replacement [1], there is still a lack of effective treatments. It is known that defects in synovial joint tissues, such as menisci, synovium, and subchondral bone, can lead to joint stress and degeneration of articular cartilage, which can result in OA [3].

Stem cell therapy and regenerative medicine approaches can be used to treat OA and repair joint cartilage defects in preclinical models [1, 4]. In early clinical trials, mesenchymal stem/stromal cell (MSC) injections have been shown to reduce pain and promote cartilage repair [4]. MSCs can be an alternative treatment for inflammatory conditions, including OA [5]. On the other hand, scaffolds play critical roles in tissue regeneration. For repairing compromised tissues, scaffolds support cell growth, proliferation, and differentiation. It is believed that scaffolds assist in cell regeneration and help create new tissues with desired shapes and properties [6, 7]. It has been demonstrated that scaffolds that provide specific microenvironmental conditions may promote MSC differentiation into a functional phenotype [8].

Scaffolds are generally classified into natural biological, synthetic, or composite natural-synthetic combinations [9, 10]. Composite scaffolds incorporating ECM components or decellularized into synthetic scaffolds have recently been developed and have shown improved biological activity [11, 12]. In this regard, the use of biological scaffolds derived from body cells, such as MSCs, has received a great deal of attention [9].

The properties of biological scaffolds can be attributed to the degradation, adaptation, and extracellular mimicry of tissues, which researchers have favored, although these scaffolds often exhibit poor mechanical properties [7, 10, 13]. Biologic scaffold materials composed of allogeneic or xenogeneic ECM are commonly used for the repair and functional reconstruction of injured and missing tissues. The transfer of signaling molecules, nutrients, and metabolic wastes is facilitated with the help of ECM-specific compounds [14].

Tissue-specific ECMs possess unique compositions and topographies and contribute to the development of individualized microenvironments within different tissue structures [15, 16]. Different components of ECM may modulate stem cell fate and function [17]. The ECM components generated by various cells, their assembly into a functional three-dimensional (3D) structure, and their involvement in tissue morphogenesis, differentiation, and physiological tissue regeneration have been the subject of extensive research over the past several decades [18–21]. Although ECM plays a pivotal role in regulating these processes, little is known about the mechanisms by which ECM produced by specific cells exerts its complex effects.

MSCs play a crucial role in tissue repair and regeneration processes, primarily because they produce ECM and extracellular vesicles containing soluble, bioactive molecules [22–24]. MSCs also contain a small population of stem and progenitor cells that are capable of differentiating into adipogenic, osteogenic, chondrogenic, and other lineages. It is presumed that the ECM produced by the stromal cell subtypes within heterogeneous populations of MSCs is at least partially responsible for regulating stromal cell functions. ECM produced by MSCs is responsible for tissue structure regeneration after injury, facilitating the migration of other cells to the injured area, and stimulating angiogenesis [25–27]. Finally, the developing ECM inhibits cell death and regulates stem and progenitor cell fate, attachment, and proliferation during tissue regeneration [28].

Decellularization of extracellular matrices is one of the most commonly used approaches for modeling a cellular microenvironment based on ECM. These matrices provide bioactive and biocompatible substances, such as matrix macromolecules and related growth factors, which often resemble the structure and composition of natural ECM microenvironments and establish the prerequisites for cell activity in vivo or in vitro [29–32]. MSCs can be cultured in 3D media using a cell layer and sheets arrangement or collagen-based systems to stimulate ECM component production. By decellularizing these 3D systems, a protein composition similar to the native ECM is obtained [33–36]. Furthermore, previous studies have shown that decellularized ECM maintains the multipotency of MSCs and hematopoietic stem cells [37, 38].

Since ECM, an essential component of stem cell microenvironments, is crucial for regulating stem cell differentiation and self-maintenance, decellularized ECM may be able to evaluate molecular communications between stem/progenitor cells and the ECM, thereby directing the role played by cell-based ECMs. The current study established an efficient decellularization protocol for a biological scaffold produced by human Wharton's jelly-derived MSCs (hWJ-MSCs) on a 3D culture collagen-based system, retaining its structure and composition. Then, we evaluated the ability of this decellularized 3D biological scaffold to restore the stem cell microenvironment in vitro and analyzed its recellularization with hWJ-MSCs, as well as its subsequent differentiation potential toward chondrocytes.

## 2. Materials and Methods

### 2.1. Isolation of MSCs Derived From Human Umbilical Cords Wharton's Jelly

Human umbilical cord samples were gathered from 10 newborn full-term infants through cesarean delivery (C-section) at Hafez and Zeinabiyyeh hospitals. Umbilical cord samples were transferred to the lab in cold phosphate-buffered saline (PBS) containing penicillin/streptomycin (100 μg/mL) (Sigma–Aldrich, St. Louis, MO, USA). Explant methods were used to isolate Wharton's jelly-derived MSCs. First, the two umbilical arteries were removed. Then, the vein was cut longitudinally and scratched. For explant culture, the umbilical cord was divided into small pieces (5 mm) and placed in a 10 cm cell culture dish, then α-MEM medium supplemented with 10% fetal bovine serum (FBS) (Gibco, Germany), penicillin/streptomycin (100 μg/mL) and 1% L-glutamine (Sigma–Aldrich) were added. The primary cells were isolated from Wharton's jelly after 14 days of culture. The completed culture medium was replaced with a fresh medium every 2 days. After MSCs reached 80% confluency, they were passaged for further use [39].

### 2.2. Characterization of hWJ-MSCs Using Flow Cytometry

The specific markers of MSCs were identified in the hWJ-MSCs population using flow cytometry. In the third passage, cells were detached and resuspended in a cold 10% FBS/PBS blocking solution for 20 min. Next, MSCs were incubated for 30 min with fluorescein isothiocyanate (FITC)-conjugated anti-CD90 and anti-CD144, and phycoerythrin-conjugated anti-CD73 and anti-CD34 (all from Abcam, Cambridge, UK). The cells were subsequently washed twice and resuspended in cold PBS. The positive or negative cell percentage was evaluated using a BD FACSCaliber flow cytometer (BD Biosciences, USA) and FlowJo, LLC (version 10.4.1) software.

### 2.3. Determining Adipogenic and Osteogenic Differentiation Potential of hWJ-MSCs

To differentiate WJ-MSCs into osteogenic and adipogenic lineages, 1 × 10^4^ cells/well at the third passage were cultured in a 24-well plate. When the cells reached about 80% confluency, the growth medium was changed to osteogenic medium (containing DMEM-LG supplemented with 10% FBS, 2 mM L-glutamine, 100 μg/mL penicillin/streptomycin, 100 nM dexamethasone, 0.2 mM L-ascorbate, and 10 mM β-glycerophosphate) or adipogenic medium (containing DMEM-LG supplemented with 10% FBS, 2 mM of L-glutamine, 100 μg/mL penicillin/streptomycin, 60 μM indomethacin, 1 μM dexamethasone, 0.5 mM of IBMX and 5 μg/mL insulin solution). hWJ-MSCs were cultured in osteogenic and adipogenic media for 28 and 21 days, respectively. The medium was changed every 3 days. Both differentiated cell lines were fixed in 4% paraformaldehyde to evaluate differentiation potential and stained with Alizarin Red S to visualize differentiated osteoblasts and Oil Red O to visualize differentiated adipocytes.

### 2.4. Preparation of 3D Biological Scaffold From hWJ-MSCs Using Collagen-Based System

The 3D biological scaffold preparation was performed as previously described [34]. A stock solution of collagen type I (3 mg/mL) extracted from rat tail (Gibco, A10483-01) was diluted with 10x DMEM (Sigma–Aldrich) at a ratio of 8:1 on ice immediately before use. hWJ-MSCs were harvested and seeded at a density of 7 × 10^5^ cells/mL of collagen. The final concentration of the collagen was 1 mg/mL. The collagen gel was polymerized for 1 h at 37°C. Then, DMEM-LG supplemented with 5% FBS was added. The cells were kept in these culture conditions for 21 days.

To evaluate cell viability and proliferation, the selected seeded scaffolds were subjected to the 3-(4,5-dimethylthiazol-2-yl)-2,5-diphenyltetrazolium bromide (MTT) assay (Sigma–Aldrich) after 1, 3, and 7 days of culture. The scaffolds were transferred into fresh wells of a 24-well plate, and 1 mg/mL MTT solution prepared in DMEM was added to each well, followed by incubation for 3 h at 37°C. Subsequently, formazan crystals formed by metabolically active cells were solubilized by adding 300 µL of dimethyl sulfoxide (DMSO; Sigma–Aldrich) for 15 min. The absorbance was then measured at 570 nm using a spectrophotometer (Epoch 2, BioTek Instruments, USA). For comparative analysis, a conventional two-dimensional (2D) monolayer culture was established on a polystyrene 24-well plate at the same initial cell seeding density as the control.

### 2.5. Decellularization Procedure

Scaffolds were collected for evaluation as either intact or decellularized scaffolds. Decellularization was performed with 1% sodium lauryl ether sulfate (SLES) (Kimia Sanaat Ataman Co., Tehran, Iran) for 48 h at a temperature of 18–20°C, utilizing a magnetic stirrer set at 100 RPM. The decellularized scaffolds were subsequently incubated in 500 U/mL deoxyribonuclease (DNase I) (Sigma–Aldrich) in PBS for 24 h, followed by treatment with 1% Triton for 1 h. Following each step, multiple rinses in PBS were conducted to eliminate cell remnants and chemical reagents. For subsequent analysis, intact or decellularized scaffolds were fixed in 10% neutral-buffered formalin, maintained at −80°C, or preserved in sterile PBS at 4°C. To inhibit microbial growth, penicillin/streptomycin was added to the solution and subsequently exposed to UV light for 20 min.

### 2.6. Confirmation of Scaffold Decellularization

#### 2.6.1. Histological Evaluation of Prepared Scaffolds

Formalin-fixed intact and decellularized scaffolds were embedded in paraffin and sectioned at 5–10 μm thickness. To determine cell removal efficiency, the sections were stained with hematoxylin and eosin (H&E) for histological evaluation. The histological slides were examined by a light microscope (Olympus BX61, Tokyo, Japan) mounted with a digital camera (Olympus DP73). Residual ECM components were qualitatively validated using Alcian blue staining for glycosaminoglycans (GAGs) and Masson trichrome staining for collagen fibers in decellularized scaffolds.

#### 2.6.2. Analysis of Residual DNA Content

The DNA quantification assay was performed using a QIAamp DNA Blood and Tissue Mini Kit (Qiagen GmbH, Hilden, Germany) according to the manufacturer's guidelines. The quantification of DNA content (ng/μL) was performed spectrophotometrically at a wavelength of 260 nm using the NanoDrop ND-1000 (Nanodrop Technologies Inc., Wilmington, DE, USA).

#### 2.6.3. Scanning Electron Microscopy (SEM) Technique

For ultrastructural evaluation, intact and decellularized scaffolds were subjected to overnight lyophilization using a Christ Alpha 2–4 LD-plus (Osterode am Harz, Germany). The samples were fixed with a 2.5% glutaraldehyde and 4% formaldehyde solution in 0.1 M PBS (pH 7.4) at 4°C overnight, followed by gradual dehydration using an increasing ethanol series. The samples were subsequently immersed in 1:2 and 2:1 hexamethyldisilazane (HMDS; Merck, Kenilworth, NJ, USA) and absolute ethanol for 20 min. After that, samples were immersed in a 100% HMDS solution overnight to facilitate air-drying in a fume hood. The samples were coated with a thin layer of gold utilizing a Q150R-ES sputter coater (Quorum Technologies, London, UK), and photomicrographs were captured using a VEGA3 microscope (TESCAN, Brno, Czech Republic) at an accelerating voltage of 10 kV.

#### 2.6.4. Confocal Raman Microscopy (CRM) Technique

Raman spectra of both intact and decellularized scaffolds were obtained. The laser power was set at 50 mW with an excitation wavelength of 785 nm. Samples were analyzed via Raman spectroscopy within the 200–2000 cm^−1^ range, achieving a resolution of 4 cm^−1^, utilizing LabSpec 6 from HORIBA Scientific.

### 2.7. Chondrogenic Differentiation of hWJ-MSCs Using Scaffold Recellularization

To assess chondrogenic differentiation, a density of 2 × 10^4^ hWJ-MSCs was cultured on a decellularized-collagen scaffold prepared as described above with or without (as a negative control) exposure to chondrogenic medium (Sigma–Aldrich) for 21 days. The medium was changed every 2 days. Cells were evaluated after 21 days of culture using the Safranin O staining to observe differentiated chondrocytes. GAGs were identified in hWJ-MSCs-derived ECM using Alcian blue staining.

#### 2.7.1. Immunohistochemical Assessment of Chondrocyte-Derived ECM Components

Expression of differentiated chondrocyte-derived ECM components, including collagen type II, aggrecan, and osteonectin, was assessed using immunohistochemistry. The fixed scaffold underwent dehydration in graded ethanol, followed by clearing in xylene, embedding in paraffin wax, and sectioning into 5 μm serial slices. The sections were positioned in a slide holder, dewaxed at 60°C for 30 min, and rehydrated. Afterward, the sections were microwaved in an unmasking antigen solution, washed with PBS, and incubated with Triton X-100 (Sigma–Aldrich) for 30 min at room temperature. To block nonspecific sites, the sections were washed with PBS and incubated with 10% goat serum for 30 min at room temperature. The sections were subsequently incubated with primary antibodies from Santa Cruz Biotechnology against collagen type II (sc-52658), aggrecan (sc-33695), and osteonectin (sc-398419) (1:100 dilution) in a wet chamber at 4°C overnight. The next morning, signals were detected employing a secondary antibody (ab6785) (goat antimouse IgG-FITC, diluted 1:200) for 40 min in the darkness. The cell nucleus was counter-stained with DAPI (Sigma–Aldrich, 5 µg/mL), followed by washing with PBS, and subsequently analyzed using a fluorescence microscope (Olympus BX51, Tokyo, Japan). A matched protocol omitting the primary antibodies incubation step served as a technical negative control.

#### 2.7.2. Quantitative Real-Time RT-PCR for Chondrogenic Markers Expression

After in vitro cultivation for 3 weeks, total RNA was extracted from the recellularized scaffolds and a conventional two-dimensional monolayer culture on a polystyrene 24-well plate with the same initial cell seeding density as the control using a standard TRIzol procedure (invitrogen) and the concentration and purity of the RNA were determined using a NanoDrop ND-2000 spectrophotometer (Thermo Fisher, USA). The mRNA was reverse-transcribed into cDNA using a cDNA synthesis kit (Add Bio Co., South Korea). The expression levels of genes encoding collagen type II alpha 1 chain (*COL2A1*), aggrecan (*ACAN*), and secreted protein acidic and cysteine-rich (*SPARC*) genes expression were quantified using the Applied Biosystems StepOne system with a high ROX SYBR Green PCR Master Mix (Add Bio Co., South Korea). Samples were quantified by the comparative 2^*−ΔΔCt*^ method with *GAPDH* as an internal control. Experiments were performed in triplicate. The oligonucleotide sequences of primers and reaction conditions used for this study are shown in Table 1.

### 2.8. Statistical Analysis

All data were shown as the mean ± standard error of the mean (SEM). After checking the data for normality using the Shapiro–Wilk test, independent samples *t*-tests were performed for DNA quantification of decellularized scaffolds and quantitative expression analysis of chondrocyte markers. One-way analysis of variance (ANOVA) followed by Tukey's multiple comparison test was used for the MTT assay data. Statistical data processing was performed using GraphPad Prism software (version 10.0, GraphPad Software, Inc., La Jolla, California, USA). Differences were considered statistically significant at the level of *p*-value < 0.05.

## 3. Results

### 3.1. Flow Cytometry-Based Characterization of hWJ-MSCs

**Figure 1:**
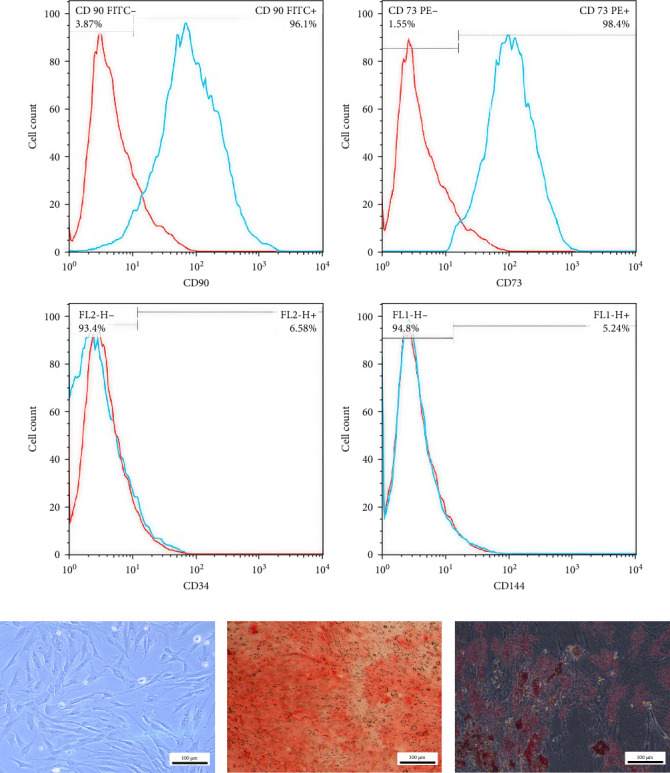
(A) Flow cytometry analysis of CD markers expression pattern of human Wharton's jelly mesenchymal stem cells (hWJ-MSCs), (B) confluent MSCs without differentiation medium, (C) osteogenic differentiation (Alizarin Red S staining), and (D) adipogenic differentiation (Oil Red O staining) (scale bar: 100 μm).

After MSCs became confluent in *α*-MEM medium without differentiation media, they were prepared for flow cytometry and marker detection. The flow cytometry results confirmed the MSC phenotype of hWJ-MSCs by expressing the surface markers of CD90 (96.1%) and CD73 (98.4%). Moreover, as expected, these cells were negative for hematopoietic stem cell (CD34, 6.58%) and endothelial cell (CD144, 5.24%) markers (Figure 1A,B).

### 3.2. Osteogenic and Adipogenic Differentiation Potential of hWJ-MSCs

After 21-day incubation in osteogenic and adipogenic medium, the osteogenesis and adipogenesis of hWJ-MSCs were assessed by the Alizarin Red S and Oil Red O staining. The presence of intracellular calcium in Alizarin Red S staining confirmed hWJ-MSCs differentiation toward osteoblasts (Figure 1C). The presence of fat droplets in the cytoplasm of the hWJ-MSCs proved adipogenic differentiation (Figure 1D).

### 3.3. MTT Assay

**Figure 2:**
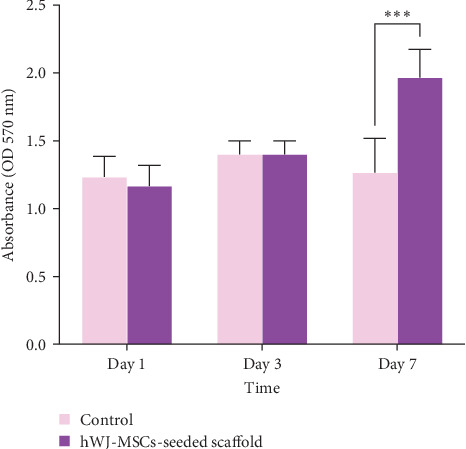
Results of MTT test for cell viability on the collagen-based biological scaffold (*n* = 3). The data are expressed as the mean ± standard error of the mean (SEM), *⁣*^*∗∗∗*^*p*=0.0003 represents a statistically significant difference.

To evaluate the cytocompatibility of the 3D biological scaffold, the viability and proliferation of hWJ-MSCs were analyzed. Results from the MTT assay confirmed that hWJ-MSCs seeded onto the 3D scaffold remained viable. A comparison of optical density (OD) values at different culture time points indicated successful cell proliferation within the scaffold. During the early phase of cultivation, cell proliferation on the collagen-based scaffold was comparable to that observed in the conventional culture system. However, significantly higher OD values were recorded in the scaffold group at later time points, suggesting an enhanced proliferation rate of hWJ-MSCs within the collagen-based 3D environment compared to the monolayer culture (Figure 2).

### 3.4. Assessment of Decellularization Efficacy

#### 3.4.1. Histological Evaluation

**Figure 3:**
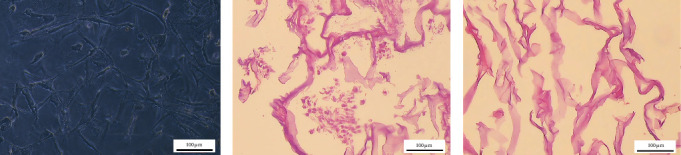
Efficacy of decellularization process (*n* = 3). (A) The cultured human Wharton's jelly mesenchymal stem cells (hWJ-MSCs) on 3D collagen scaffolds with a star-like morphology, (B) hematoxylin and eosin (H&E) micrographs of an intact scaffold containing hWJ-MSCs, and (C) decellularized scaffold (scale bar: 100 μm).

The efficacy of the decellularization process was evaluated using H&E staining and analysis of residual DNA content. The decellularized scaffolds qualitatively showed no trace of nucleic materials compared to intact ones (Figure 3A–C).

**Figure 4:**
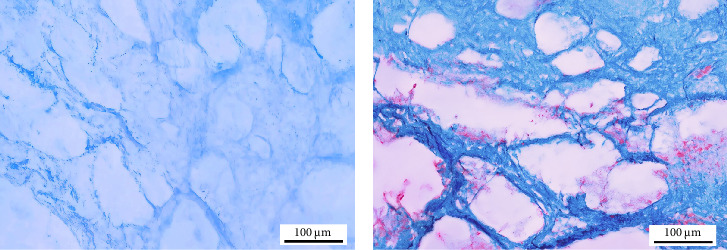
Qualitative assessment of residual extracellular matrix structures in the decellularized scaffold (*n* = 3). (A) Alcian blue staining for glycosaminoglycans (GAGs) and (B) Masson trichrome staining for collagen fibers (scale bar: 100 μm).

Alcian blue and Masson trichrome staining revealed the continued presence of GAGs and collagen fibers, respectively (Figure 4A,B).

#### 3.4.2. DNA Quantification

DNA quantification analysis revealed a significant decrease in DNA content following decellularization, with values of 70.21 ± 7.62 ng/mg dry tissue weight in decellularized scaffolds compared to 1253.22 ± 56.34 ng/mg dry tissue weight in intact samples (*n* = 3, *p*  < 0.0001).

#### 3.4.3. SEM

**Figure 5:**
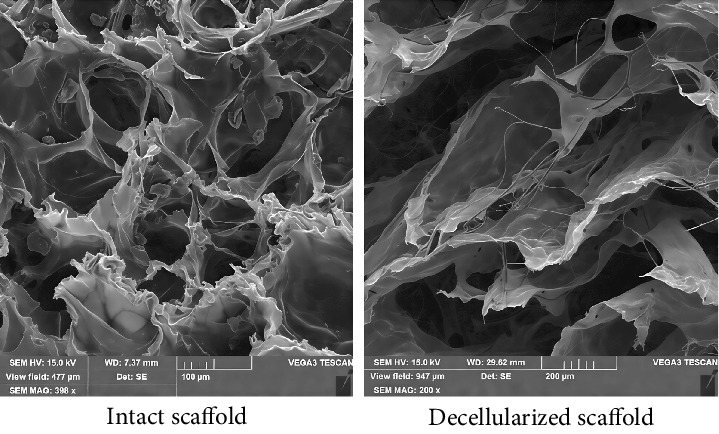
Scanning electron microscopy (SEM) images of the intact and decellularized scaffold (*n* = 3). SEM assessment revealed that the ultrastructure of the decellularized scaffold was devoid of cells using the SLES 1% protocol after decellularization. SLES: sodium lauryl ether sulfate.

The integrity and efficiency of the decellularization process were confirmed by SEM. The decellularized scaffolds showed no trace of cells compared to intact ones. Consistent with light microscopy findings, SEM demonstrated that the orientation and structures of the collagen fibers remained essentially unchanged. In lower and higher magnifications, decellularized scaffolds showed a porous structure with complex fibers and an ECM network (Figure 5).

#### 3.4.4. Raman Spectrum

Both intact and decellularized scaffolds displayed similar Raman spectra patterns after normalization and baseline correction. Peaks at 418 and 538 cm^−1^ were more likely associated with collagen-related vibrations; peaks at 519 and 576 cm^−1^ signify the phosphatidylinositol, and at 759 and 1298 cm^−1^ represent proteins and collagen-related peaks, as they fall within the typical range for amide I and III bands, respectively [40]. Upon decellularization, all these bands showed a marked reduction in intensity, indicating that the protocol successfully reduced the lipid and protein content.

**Figure 6:**
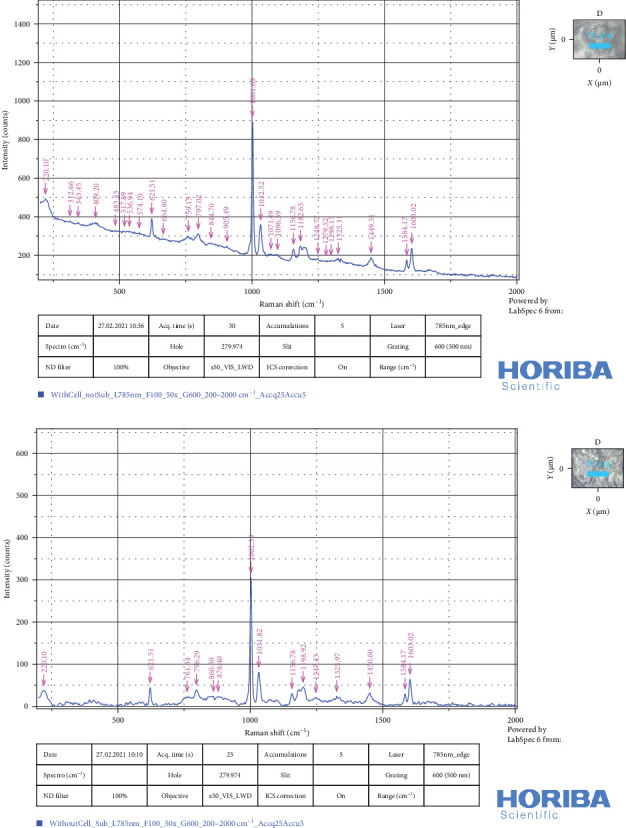
(A) Raman spectra of intact (with cells) and (B) decellularized scaffolds (without cells) (*n* = 3). Laser power level = 50 mW, excitation laser wavelength = 785 nm, Raman spectra range = 200–2000 cm^−1^, and resolution = 4 cm^−1^ (powered by LabSpec 6 from HORIBA Scientific).

Vibration at 840–60 cm^−1^ can represent polysaccharide structure. A peak at 907 cm^−1^ represents an unknown mode. 1071 cm^−1^ represents GAGs. Bands allocated for protein were also detected. Specific bands for C–H, C–C, and C–N stretching vibration at 1449, 1450, and 1156 cm^−1^ were observed. Vibrations at 1200–1300 cm^−1^ determined amide III, and a peak at 879 cm^−1^ belonged to hydroxyproline content (collagen). The resonances at 1031 and 1032 cm^−1^ were indicative of the carbohydrate components found in the ECM, including GAGs and hyaluronan. They can be assigned to CH_2_-CH_3_ bending modes of collagen and phenylalanine/proline (collagen assignment). In addition, bands at 621, 1000, 1002, 1583, and 1603 cm^−1^ indicate the presence of phenylalanine in the collagen. Peaks for tryptophan, cytosine, and guanine, indicating the presence of DNA, can be found at 666, 759, 1096, 1184, 1247, 1279, and 1325 cm^−1^ [40]. Comparing the Raman spectra of intact and decellularized scaffolds revealed a significant reduction in carbohydrate, lipid, and DNA content (Figure 6A,B).

### 3.5. Chondrogenic Differentiation of hWJ-MSCs

**Figure 7:**
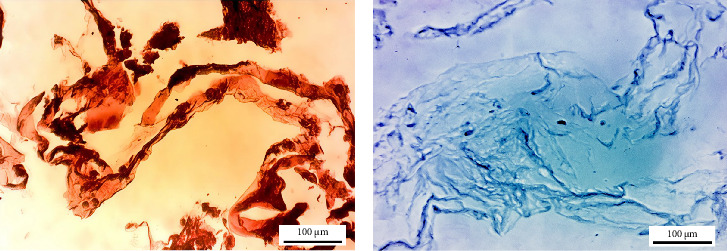
Qualitative assessment of extracellular matrix structures in the recellularized scaffold (*n* = 3). (A) Safranin O staining is used to determine hWJ-MSCs differentiation into chondrocytes and (B) Alcian blue staining is used to determine GAGs in the produced ECM (scale bar: 100 μm).

The differentiation of hWJ-MSCs on the 3D scaffold was detected only in the presence of a chondrogenic medium; the hWJ-MSCs were differentiated into chondrocytes, and the intensity of Safranin O and Alcian blue staining, demonstrating increases in GAGs and ECM by day 21 (Figure 7A,B).

#### 3.5.1. Immunohistochemical Data

**Figure 8:**
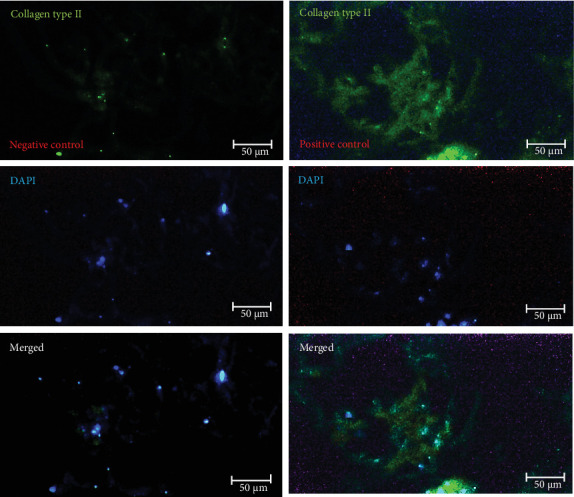
In vitro analysis of chondrocyte marker of collagen type II immunoreactivity on recellularized scaffolds treated with chondrogenic medium (*n* = 3) (scale bar: 50 µm). The photomicrographs in the first row were stained without (negative control) or with (positive control) a collagen type II primary antibody. Cell nuclei in the recellularized scaffolds are stained with DAPI, as shown in the middle photomicrographs. The third row is a merged image of the first and second rows.

**Figure 9:**
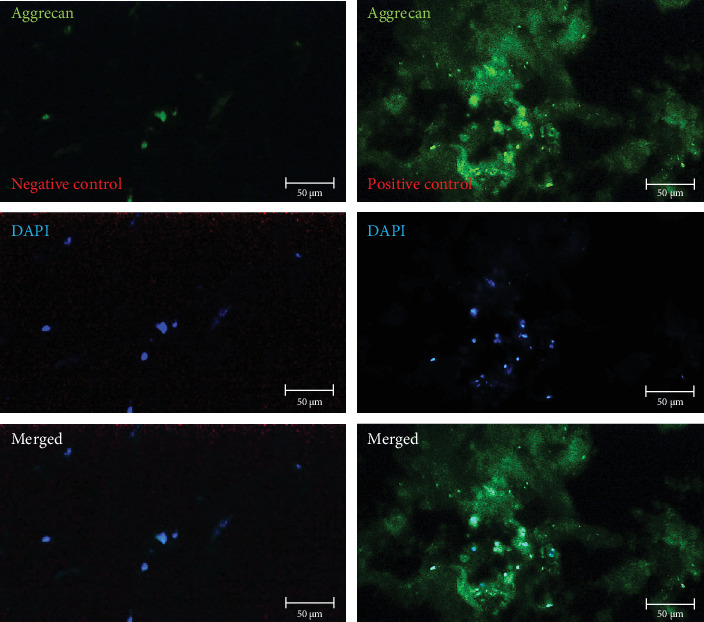
In vitro analysis of chondrocyte marker of aggrecan immunoreactivity on recellularized scaffolds treated with chondrogenic medium (*n* = 3) (scale bar: 50 µm). The photomicrographs in the first row were stained without (negative control) or with (positive control) an aggrecan primary antibody. Cell nuclei in the recellularized scaffolds are stained with DAPI, as shown in the middle photomicrographs. The third row is a merged image of the first and second rows.

**Figure 10:**
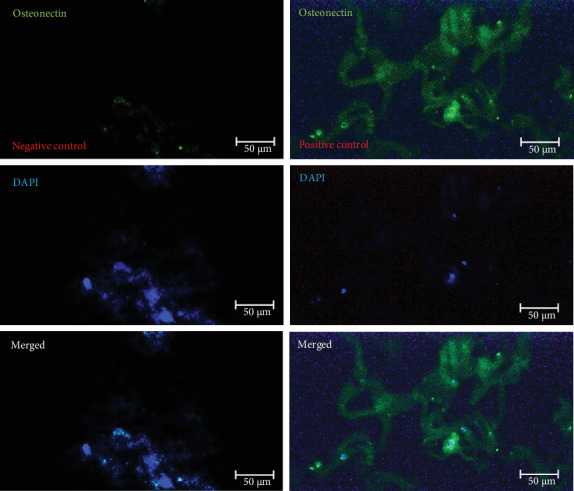
In vitro analysis of chondrocyte marker of osteonectin immunoreactivity on recellularized scaffolds treated with chondrogenic medium (*n* = 3) (scale bar: 50 µm). The photomicrographs in the first row were stained without (negative control) or with (positive control) an osteonectin primary antibody. Cell nuclei in the recellularized scaffolds are stained with DAPI, as shown in the middle photomicrographs. The third row is a merged image of the first and second rows.

After chondrogenic differentiation of hWJ-MSCs, collagen type II, aggrecan, and osteonectin production were evaluated. Immunoreactivity analysis of expanded cells showed that they could significantly express specific chondrocyte markers, including collagen type II, aggrecan, and osteonectin, after 21 days in the presence of a chondrogenic medium (Table 2). In vitro expression analysis of these three chondrocyte markers (collagen type II, aggrecan, and osteonectin) on scaffolds compared to negative controls is shown in Figures 8–10, respectively.

#### 3.5.2. Gene Expression Data

**Figure 11:**
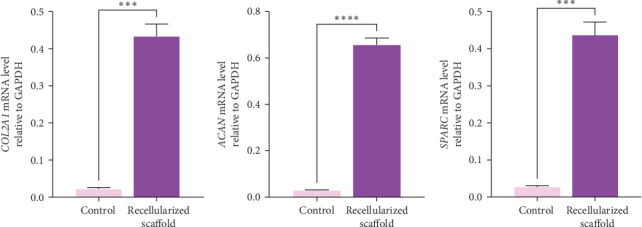
Relative expression of chondrocyte-associated genes (A) *COL2A1*, (B) *ACAN*, and (C) *SPARC* in recellularized scaffolds with hWJ-MSCs treated with chondrogenic medium (*n* = 3). Data are expressed as the mean ± standard error of the mean (SEM), *⁣*^*∗∗∗*^*p*=0.0003 and *⁣*^*∗∗∗∗*^*p*  < 0.0001 represent significant differences.

The relative expression of the chondrocyte-associated genes *COL2A1*, *ACAN*, and *SPARC* was assessed by qRT-PCR analysis in hWJ-MSCs cultured on scaffolds in the absence and presence of chondrogenic medium. Our results demonstrate a statistically significant upregulation of these genes in hWJ-MSCs cultured on the decellularized scaffold in the presence of chondrogenic medium compared to negative controls (*p*  < 0.001), confirming successful chondrogenic differentiation at the transcriptional level (Figure 11).

## 4. Discussion

In the current study, we have evaluated the ability of decellularized collagen scaffolds to induce differentiation of hWJ-MSCs into chondrocytes in vitro. The scaffold is composed of type I collagen, has demonstrated high biocompatibility, and can support host cell proliferation and differentiation. Chondrogenic differentiation was evaluated using immunogenic cartilage-specific markers, including type II collagen, aggrecan, and osteonectin. The results demonstrated that all chondral markers showed higher expression on the decellularized scaffold in the presence of a conditioning medium, and there were statistically significant differences compared to the negative control. In this study, we found that the decellularized biological scaffold can induce chondrogenesis of hWJ-MSCs in vitro and is a potential candidate for cartilage repair.

In this regard, similar results were reported; Ghiasi et al. demonstrated that inherent fibrin glue scaffolds could induce the differentiation of MSCs to chondrocytes. Their differentiation into cartilage was evaluated by assessing the expression of aggrecan, types I and II collagen, and Sox9 mRNAs. Their results showed that the expression of these markers increased compared to alginate [41]. Furthermore, the tricalcium phosphate-collagen-hyaluronate (TCP-COL-HA) scaffold plays a crucial role in the rabbit MSCs' chondrogenesis induction and repair of osteochondral defects in vitro and in vivo. The chondrogenic markers, including aggrecan and three types of collagens (COL I, II, and X), had higher expression in the TCP-COL-HA group than those in the TCP-COL group [42]. The results of Larson et al. indicated that 3D woven poly (ε-caprolactone; PCL) scaffolds support cellularization as well as ECM synthesis, accumulation, and remodeling in vivo. The precultured-MSCs-PCL group maintained high levels of chondrogenic markers from week 0 to week 8 [43]. A porous scaffold derived from articular cartilage and a nanofibrous Wharton's jelly scaffold had the ability to induce chondrogenic differentiation of adipose-derived adult stem cells without the need for exogenous growth factors. These findings support the potential of a processed cartilage ECM as a biomaterial scaffold for cartilage tissue engineering [44, 45]. Overall, these results, in agreement with our findings, suggest that the chondrogenic differentiation of MSCs in the presence of decellularized scaffolds could act as replacement therapy for articular cartilage defects.

Articular cartilage is a highly organized tissue characterized by zonal heterogeneity of cells, protein components of the ECM, and fibril orientations, all of which determine its depth-dependent mechanical properties [46]. Ideally, a scaffold should possess architectural, physicochemical, biological, and mechanical characteristics [47, 48]. The materials used in cartilage tissue engineering scaffolds primarily fall into two categories: natural and synthetic materials. Despite their excellent biocompatibility, ease of degradation, relatively low toxicity, ease of absorption, and low inflammatory reactions, natural materials also have poor mechanical strength and are difficult to control [7, 49]. Many researchers have focused on the decellularized matrix for constructing cartilage tissue-engineered scaffolds [50, 51].

The development and application of decellularized ECM matrices have rapidly expanded in recent years to provide therapeutic goals in tissue engineering and regenerative medicine [36, 52–54]. The cell source producing ECM plays a fundamental role in determining its functional characteristics [55]. MSCs are a suitable source for cell therapy and tissue engineering purposes compared to other cells due to their multipotency [22, 56]. hWJ-MSCs are easily accessible, noncontroversial sources of MSCs with self-renewal ability and extended proliferation potential, making them excellent candidates for tissue engineering [57]. Decellularized ECM produced by MSCs has recently been identified as a suitable substrate for better cell development [29, 58, 59]. MSCs cultured on these scaffolds better maintain their proliferation, phenotype, and differentiation potential [36, 60]. However, the mechanisms of these effects and the behavior of different MSC sources remained poorly understood.

It has been demonstrated that MSCs-derived ECM contains specific molecules and mechanical signals that influence tissue regeneration and cellular behavior [61]. ECM is definitely a 3D network of extracellular macromolecules comprising collagen, enzymes, and glycoproteins. Collagen, a vital component of the ECM, contributes to the structural and biochemical support of its surrounding cells [62]. Proteins such as collagen and ECM growth factors provide substrates for cellular proliferation, adhesion, migration, polarity, differentiation, and apoptosis [63]. Moreover, previous studies similar to the present study have shown that a 3D-culture system using collagen provides better conditions for MSC growth and differentiation [34, 64, 65].

Decellularized scaffolds offer several advantages over artificial synthetic biomaterials, including the ability to obtain a naturally designed architecture, retain the inherent growth factors that encourage cellular growth, and restore organ function [66]. By facilitating cellular attachment, increasing proliferation, and providing a 3D nontoxic structure, decellularized scaffolds are ideal for regenerative medicine [67–70].

The current study utilized a 1% SLES agent to decellularize the collagen-based 3D biological scaffold derived from hWJ-MSCs. The decellularized scaffold underwent comprehensive characterization to assess its suitability for cartilage tissue engineering. Histological analyses, including H&E staining, confirmed the effective removal of cellular nuclei, indicating successful decellularization. SEM revealed a porous and interconnected microstructure essential for nutrient diffusion and cell infiltration. Biochemical assays demonstrated the retention of key ECM components, such as GAGs and collagen, which are critical for maintaining the mechanical properties and bioactivity of the scaffold. Upon recellularization with hWJ-MSCs, the scaffold exhibited a conducive environment for chondrogenic differentiation. Immunohistochemical and transcriptional assessments revealed the upregulation of cartilage-specific markers, including type II collagen, aggrecan, and osteonectin. These findings suggest that the bioactive cues present in the MSC-derived ECM, combined with the structural support of type I collagen, synergistically promote chondrogenesis.

The dual composition of the scaffold plays a pivotal role in its functionality. Type I collagen provides mechanical strength and structural framework, facilitating cell attachment and proliferation. Simultaneously, the MSC-derived ECM contributes bioactive molecules that guide stem cell differentiation toward the chondrogenic lineage. This combination mirrors the native cartilage microenvironment, enhancing the scaffold's efficacy in cartilage regeneration applications. Our findings are consistent with previous studies that have demonstrated the potential of decellularized ECM scaffolds in supporting chondrogenesis. For instance, Zhang et al. [12] reviewed that decellularized cartilage scaffolds could promote the differentiation of MSCs into chondrocytes, highlighting the importance of preserving ECM components during decellularization. Additionally, the retention of GAGs and collagen within the scaffold has been shown to be crucial for maintaining its biomechanical properties and facilitating cell-matrix interactions.

Based on all detected changes in hWJ-MSCs cultured on the biological 3D collagen-based decellularized scaffold, we suggest that hWJ-MSCs-generated ECM-mediated signaling, by which the cells can rapidly and efficiently differentiate into chondrocytes. It is noteworthy that hWJ-MSCs implanted in the prepared biological scaffolds should be capable of recognizing specific external signals. Therefore, we hypothesize that ECM generated by hWJ-MSCs facilitates the response of target cells to specific differentiation stimuli (e.g., chondrogenic medium) by converting them into a competent state. There is still a need to investigate whether these effects are specific to various cultured MSCs or can also be manifested in vivo.

Despite the promising outcomes demonstrated in this study, several limitations should be acknowledged. First, while the in vitro environment provided valuable insights into the chondrogenic differentiation potential of hWJ-MSCs on the decellularized collagen-based scaffold, the functional formation of mature cartilage tissue was not directly assessed. Future in vivo studies are necessary to evaluate critical features such as cartilage zonation, integration with host tissue, and long-term stability and functionality of the regenerated constructs. Second, the biomechanical properties of the regenerated ECM were not evaluated in the current study due to limitations in instrumentation availability. Mechanical characterization, including assessments of compressive modulus, elasticity, and viscoelastic properties using techniques such as uniaxial compression testing and dynamic mechanical analysis, will be essential in future investigations to comprehensively validate the scaffold's mechanical suitability for cartilage repair applications. By addressing these limitations in future work, we aim to further strengthen the translational potential of our scaffold platform for clinical cartilage regeneration.

## 5. Conclusion

The ECM produced by MSCs is crucial in shaping the cellular microenvironment and influencing cell fate, particularly within stem cell niches. Our biological 3D collagen-based scaffold decellularization method enabled an examination of the role of hWJ-MSCs-derived ECM in the proliferation and differentiation of multipotent cells. The biological scaffold produced by hWJ-MSCs is able to largely reestablish the native matrix composition and structure. The in vitro simulation of stem cell niche apparatuses may ensure the effective response of multipotent progenitor cells to external chondrogenic differentiation stimuli. Obtaining 3D collagen-based scaffolds from hWJ-MSCs represents a promising in vitro model for determining the contribution of ECM to specific cell microenvironments under various conditions. In addition, our results may also be useful in the development of new biomedical cell-free products for regenerative medicine based on decellularized ECM.

## Figures and Tables

**Table 1:** Studied genes, primer sequences, and PCR conditions.

Transcripts	Primer	Primer sequences (5′–3′)	Thermocycling condition
*COL2A1*	Forward	CACGCTCAAGTCCCTCAACA	95°C/10 min, 40 cycles at 95°C/15 s, 58°C/20 s, and 72°C/30 s
	Reverse	TCTATCCAGTAGTCACCGCTCT	

*ACAN*	Forward	GGAGGAGCAGGAGTTTGTCAA	95°C/10 min, 40 cycles at 95°C/15 s, 60°C/20 s, and 72°C/30 s
	Reverse	TGTCCATCCGACCAGCGAAA	

*SPARC*	Forward	GCAAAGGGAAGTAACAGACAC	95°C/10 min, 40 cycles at 95°C/15 s, 59°C/20 s, and 72°C/30 s
	Reverse	GAAAGGTAA AGGAGGAAATGG	

*GAPDH*	Forward	CAAGAAGGTGGTGAAGCAGG	95°C/10 min, 40 cycles at 95°C/15 s, 59°C/20 s, and 72°C/30 s
	Reverse	CACTGTTGAAGTCGCAGGAG	

*Note:* Collagen type II alpha 1 chain (*COL2A1*), aggrecan (*ACAN*), secreted protein acidic and cysteine rich (*SPARC*), and glyceraldehyde-3-phosphate dehydrogenase (*GAPDH*).

**Table 2:** In vitro quantitative expression analysis of chondrocyte markers (collagen type II, aggrecan, and osteonectin) in differentiated cells on scaffolds.

	Chondrogenic differentiation markers		
	Collagen type II	Aggrecan	Osteonectin
Treated cells	47.37 ± 1.43	68.00 ± 2.51	34.62 ± 1.95
Control	2.20 ± 0.50	3.70 ± 0.89	2.89 ± 0.91
*p*-Value	<0.0001	<0.0001	<0.0001

*Note:* The data were expressed as the mean ± SEM (*n* = 3).

## Data Availability

The data that support the findings of this study are available from the corresponding author upon reasonable request.
